# Crystal and mol­ecular structure of aflatrem

**DOI:** 10.1107/S2056989015019040

**Published:** 2015-10-17

**Authors:** Bruno N. Lenta, Jules Ngatchou, Patrice T. Kenfack, Beate Neumann, Hans-Georg Stammler, Norbert Sewald

**Affiliations:** a Department of Chemistry, Higher Teacher Training College, University of Yaoundé 1, PO Box 47, Yaoundé, Cameroon; b Department of Organic Chemistry, University of Yaoundé 1, PO Box 812, Yaoundé, Cameroon; c Department of Inorganic Chemistry, University of Yaoundé 1, PO Box 812, Yaoundé, Cameroon; d Department of Chemistry, University of Bielefeld, PO Box 100131, 33501 Bielefeld, Germany

**Keywords:** crystal structure, aflatrem, indole-diterpenoid, fungal endophytes, *Aspergillus* species, N—H⋯*Cg* (indole), hydrogen bonding

## Abstract

The crystal structure of the title compound, C_32_H_39_NO_4_, confirms the absolute configuration of the seven chiral centres in the mol­ecule. The molecule has a 1,1-dimethylprop-2-enyl substituent on the indole nucleus and this nucleus shares one edge with the five-membered ring which is, in turn, connected to a sequence of three edge-shared fused rings. The skeleton is completed by the 7,7-trimethyl-6,8-dioxabi­cyclo­[3.2.1]oct-3-en-2-one group connected to the terminal cyclohexene ring. The two cyclohexane rings adopt chair and half-chair conformations, while in the dioxabi­cyclo­[3.2.1]oct-3-en-2-one unit, the six-membered ring has a half-chair conformation. The indole system of the mol­ecule exhibits a tilt of 2.02 (1)° between its two rings. In the crystal, O—H⋯O hydrogen bonds connect mol­ecules into chains along [010]. Weak N—H⋯π inter­actions connect these chains, forming sheets parallel to (10-1).

## Related literature   

For background to indole diterpenoids from endophytes, see: Strobel & Daisy (2003 ▸); Munday-Finch *et al.* (1996 ▸); Gallagher *et al.* (1980*a* ▸,*b* ▸); Lenta *et al.* (2007 ▸); Phongpaichit *et al.* (2007 ▸). For studies of *Aspergillus sp*, see: Nicholson *et al.* (2009 ▸); Duran *et al.* (2006 ▸). For the pharmacological basis of the behavioural effects of this mol­ecule, see: Tinao-Wooldridge *et al.* (1995 ▸). For the isolation of fungal endophytes from the stem of *Symphonia globulifera*, see: Petrini *et al.* (1992 ▸); Amin *et al.* (2014 ▸). For geometric details of indole compounds, see: Krishna *et al.* (1999 ▸). For circular dichroism experiments on the title compound, see: Sun *et al.* (2014 ▸). For information on the Cambridge Structural Database (CSD), see: Groom & Allen (2014 ▸).
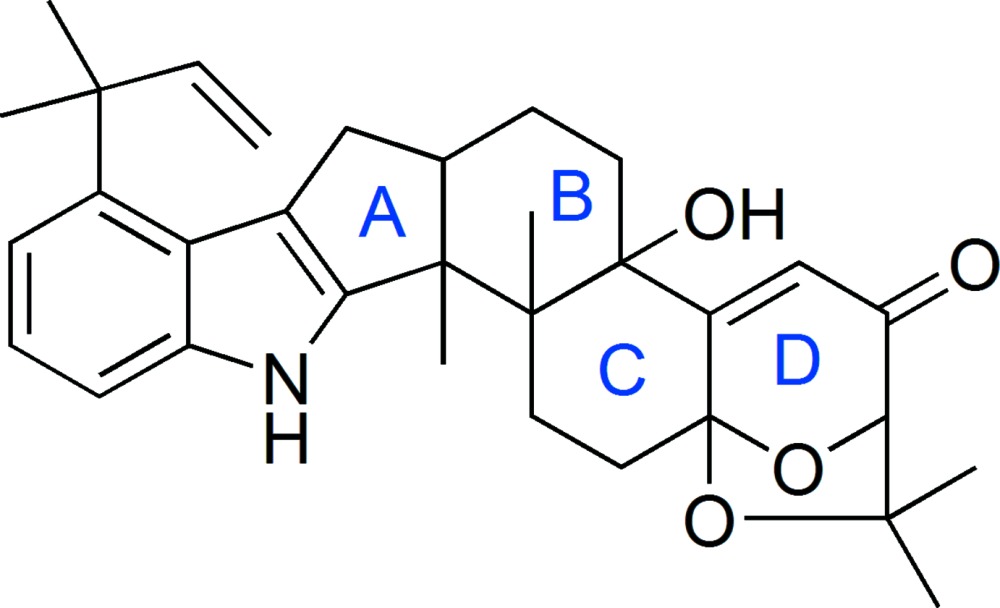


## Experimental   

### Crystal data  

C_32_H_39_NO_4_
*M* *_r_* = 501.64
Monoclinic, 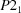

*a* = 12.8022 (5) Å
*b* = 6.4019 (2) Å
*c* = 15.9557 (6) Å
β = 98.821 (4)°
*V* = 1292.24 (9) Å^3^
*Z* = 2
Cu *K*α radiation
μ = 0.66 mm^−1^
*T* = 100 K
0.18 × 0.14 × 0.02 mm

### Data collection  

Agilent SuperNova Dual Source diffractometer with an Atlas detector
Absorption correction: gaussian (*CrysAlis PRO*; Agilent, 2013 ▸) *T*_min_ = 0.899, *T*_max_ = 1.000
19981 measured reflections
4585 independent reflections
4078 reflections with *I* > 2σ(*I*)
*R*_int_ = 0.050

### Refinement  

*R*[*F* ^2^ > 2σ(*F* ^2^)] = 0.056
*wR*(*F* ^2^) = 0.150
*S* = 1.06
4585 reflections
341 parameters
1 restraint
H-atom parameters constrained
Δρ_max_ = 0.35 e Å^−3^
Δρ_min_ = −0.23 e Å^−3^
Absolute structure: Flack *x* determined using 1671 quotients [(*I* ^+^)−(*I* ^−^)]/[(*I* ^+^)+(*I* ^−^)] (Parsons & Flack, 2004 ▸)
Absolute structure parameter: 0.09 (14)

### 

Data collection: *CrysAlis PRO* (Agilent, 2013 ▸); cell refinement: *CrysAlis PRO*; data reduction: *CrysAlis PRO*; program(s) used to solve structure: *SHELXS97* (Sheldrick, 2008 ▸); program(s) used to refine structure: *SHELXL97* (Sheldrick, 2008 ▸); molecular graphics: *DIAMOND* (Brandenburg, 1999 ▸); software used to prepare material for publication: *OLEX2* (Dolomanov *et al.*, 2009 ▸).

## Supplementary Material

Crystal structure: contains datablock(s) global, I. DOI: 10.1107/S2056989015019040/lh5789sup1.cif

Structure factors: contains datablock(s) I. DOI: 10.1107/S2056989015019040/lh5789Isup2.hkl

**Fig. 1.**
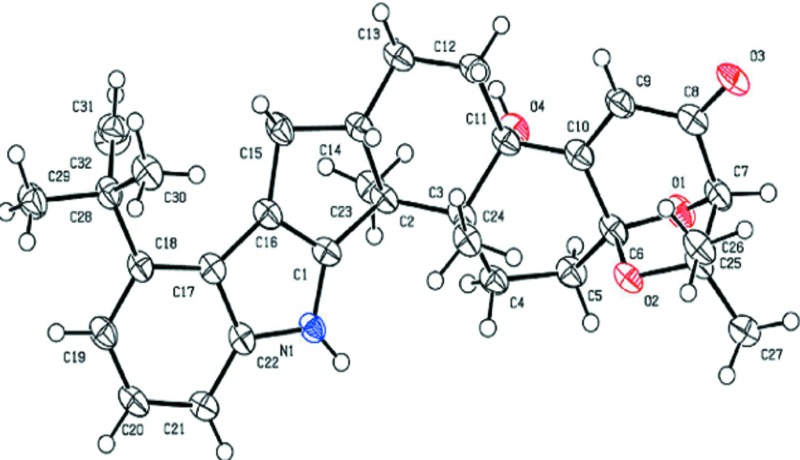
The molecular structure of aflatrem with the atom-labelling scheme. Displacement ellipsoids are drawn at the 50% probability level.

. DOI: 10.1107/S2056989015019040/lh5789fig1.tif
The mol­ecular structure of aflatrem with the atom-labelling scheme. Displacement ellipsoids are drawn at the 50% probability level.

**Fig. 2.**
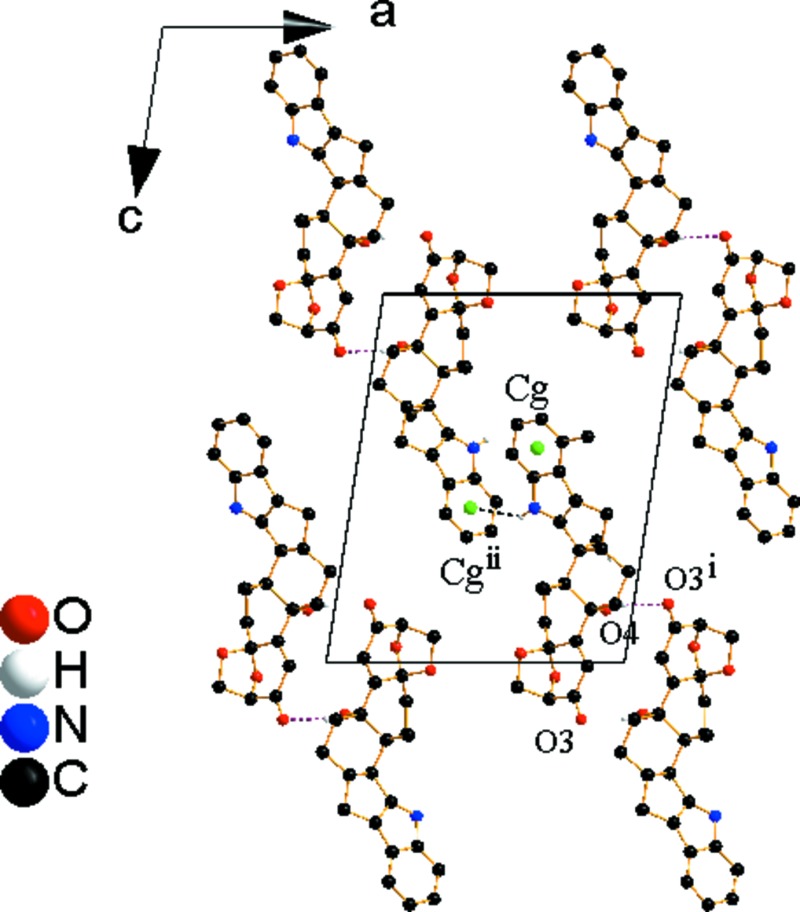
Crystal packing of aflatrem showing O—H···O hydrogen-bonded (dashed lines) zigzag chains along the b axis in the (010) plane . Weak N—H···π interactions are also shown as dashed lines. Symmetry codes: (i) - x+ 2, y - 1/2, -z + 2; (ii) -x + 1, y - 1/2, -z + 1; (iii) -x + 1, y + 1/2, -z + 1; (iv) -x + 2, y + 1/2, -z + 2.

Click here for additional data file.b x y z x y z x y z x y z . DOI: 10.1107/S2056989015019040/lh5789fig2.tif
Crystal packing of aflatrem showing O—H⋯O hydrogen-bonded (dashed lines) zigzag chains along the *b* axis in the (010) plane. Weak N—H⋯π inter­actions are also shown as dashed lines. Symmetry codes: (i) − *x* + 2, *y* − 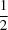, −*z* + 2; (ii) −*x* + 1, *y* − 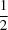, −*z* + 1; (iii) −*x* + 1, *y* + 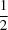, −*z* + 1; (iv) −*x* + 2, *y* + 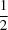, −*z* + 2.

**Fig. 3.**
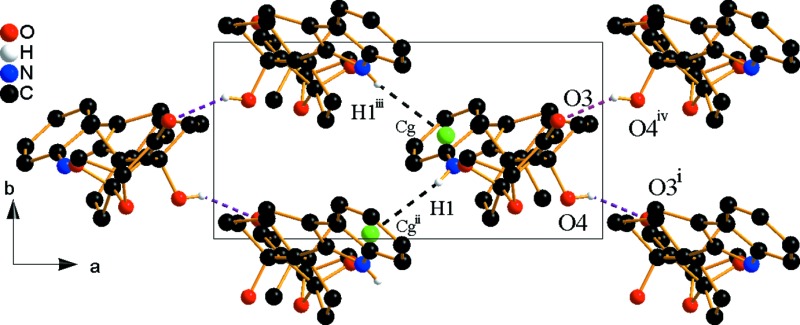
Crystal packing of aflatrem showing O—H···O hydrogen-bonded (dashed lines) zigzag chains along the b axis in the (001) plane. Weak N—H···π interactions are also shown as dashed lines. Symmetry codes: (i) -x + 2, y - 1/2, -z +2; (ii) -x + 1, y - 1/2, -z + 1; (iii) -x + 1, y + 1/2, -z + 1 and (iv) -x + 2, y + 1/2, -z + 2.

Click here for additional data file.b x y z x y z x y z x y z . DOI: 10.1107/S2056989015019040/lh5789fig3.tif
Crystal packing of aflatrem showing O—H⋯O hydrogen-bonded (dashed lines) zigzag chains along the *b* axis in the (001) plane. Weak N—H⋯π inter­actions are also shown as dashed lines. Symmetry codes: (i) −*x* + 2, *y* − 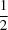, −*z* + 2; (ii) −*x* + 1, *y* − 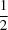, −*z* + 1; (iii) −*x* + 1, *y* + 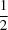, −*z* + 1 and (iv) −*x* + 2, *y* + 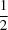, −*z* + 2.

CCDC reference: 1430332

Additional supporting information:  crystallographic information; 3D view; checkCIF report

## Figures and Tables

**Table 1:** Hydrogen-bond geometry (, ) *Cg* is the centroid of the C17C22 ring.

*D*H*A*	*D*H	H*A*	*D* *A*	*D*H*A*
O4H4O3^i^	0.82	2.03	2.757(3)	148
N1H1*Cg* ^ii^	0.86	2.78	3.527(1)	146

Symmetry codes: (i) 
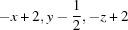
; (ii) 
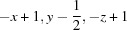
.

## supplementary crystallographic information

### S1. Comment

The search of compounds from plant endophytes has been the subject of research interest (Strobel & Daisy 2003). They produce a plethora of substances with potential applications in agriculture, medicine, pharmaceutical and for industry (Petrini *et al.* 1992; Strobel & Daisy, 2003; Phongpaichit *et al.*, 2007). We are interested in the isolation and the structural study of compounds produced by endophytes from Cameroonian medicinal plants with pharmacological properties (Lenta *et al.*, 2007) and one of the compounds that we have isolated from the fungal endophyte of the stem of Symphonia globulifera was aflatrem. Some authors have identified the biosynthetic genes of this molecule in Aspergilius sp (Nicholson *et al.*, 2009; Duran *et al.*, 2006). We report herein the study of its molecular and crystal structure.

Aflatrem crystallizes in the non-centrosymmetric space group *P*2_1_ and its asymmetric unit consists of a single molecule as shown in Figure 1. As known in the literature, the molecule has a 1,l-dimethyl-2-propenyl substituent on the indole nucleus at position C18 and this nucleus shares one edge with the 5-membered ring (A) belonging to a group of three fused rings like an anthracene system (see Fig.1). The two others rings (6-membered, B and C) also share one edge with the 7, 7-trimethyl-6,8- dioxabicyclo[3.2.1]oct-3-en-2-one group. All the bond distances observed in the compounds are in agreement with the bonds distance of the Cambridge Structural Database (CSD, Groom & Allen, 2014). In the indole ring system, a small tilt of 2.02 (1)° is observed between the 6 and 5-membered rings. This value as well as the values of the bond angles is near the values always obtained in the indole based compounds (Krishna *et al.*, 1999). The ring A (C1/C16/C15/C14/C2) adopts a dihedral angle of 3.12 (1)° with the 5-membered ring of indole system. With the exception of the C1–C2–C14 angle (96.9 (1)°),the values of the bond angles in this ring are in the range between 100 and 112° and they are in good agreement with the ideal conformation for which the angle is 107°. The lower value observed could be favored by the chair conformation of the B ring which shares one edge (C2 and C14) with ring A. This conformation is close to the ideal chair conformation since the bond angles range from 107 to 113° compared to an ideal value of 109°. The ring C assumes a half-chair conformation. The bicyclo[3.2.1]oct-3-en-2-one system is composed by a 6-membered ring named D (C6/C10/C9/C8/C7/O1) sharing one edge (C6 –C7) with a 5-membered ring called E (O1/C7/C6/O2/C25). The carbon atoms of ring D lie in the same plane and the O1 atom is located at 0.80 (1) Å from this plane. This atom is also located at 0.656 (1) Å from the plane which contains the carbon atoms of ring E and the dihedral angle between the two planes is 69.07 (1)°. The methyl and hydroxyl groups linked to the fused ring give the absolute configuration of 1S,3*R*,6S,7*S*,11*R*,12*S*,13S determined by Cu Kα*X*-radiation with the Flack parameter being refined to 0.09 (14) and this configuration is in agreement with the previous circular dichroism assignment reported by Sun *et al.* (2014).

The crystal packing of the aflatrem molecules is illustrated in Figs. 2 and 3. In the crystal, molecules are connected along the *b* axis via O—H···O hydrogen bonds. In addition, weak N—H···π(indole) interactions connect these chains forming planes parallel to (10-1). This N—H···π(indole) interaction is typical of indole-based molecules as reported by Krishna *et al.* (1999).

### S2. Experimental

The isolation of fungal endophytes from the stem of *Symphonia globulifera* was carried out aat the University of Yaoundé 1 (Cameroon) and was based on the method described by Petrini *et al.* (1992). One of the fungi was identified to *Aspergillus sp*. according to the method described by Amin *et al.* (2014) and cultured in solid medium prepared from 1 kg of rice distributed in the glass flask (total capacity of 2.5 *L*) at a rate of 200 g of rice in 200 ml of distilled water. After one month of incubation at 301K in the same laboratory, the culture medium was extracted with EtOAc and the extract concentrated on a rotary evaporator under vacuum at a temperature of 313K to yield 20.1 g of extract. This extract was subjected to column chromatography (CC) over silica gel (0.023–0.20 mesh, Merck) and eluted with a gradient system of petroleum ether /ethyl acetate to afford aflatrem (7.5 mg). The colourless crystals obtained were sent to the Laboratory of Inorganic and Structural Chemistry at Bielefeld University (Germany) for X-ray diffraction measurements.

### S3. Refinement

H atoms were placed in calculated positions with C–H = 0.93-0.98Å, N—H = 0.86Å and O—H = 0.82Å. They were included in calculated positions with U_iso_(H) = 1.2U_eq_(C) or 1.5U_eq_(C_methyl_,O).

### Crystal data

**Table:**

C_32_H_39_NO_4_	*F*(000) = 540
*M**_r_* = 501.64	*D*_x_ = 1.289 Mg m^−^^3^
Monoclinic, *P*2_1_	Cu *K*α radiation, λ = 1.5418 Å
*a* = 12.8022 (5) Å	Cell parameters from 6775 reflections
*b* = 6.4019 (2) Å	θ = 4.8–66.4°
*c* = 15.9557 (6) Å	µ = 0.66 mm^−^^1^
β = 98.821 (4)°	*T* = 100 K
*V* = 1292.24 (9) Å^3^	Plate, colourless
*Z* = 2	0.18 × 0.14 × 0.02 mm

### Data collection

**Table:**

Agilent SuperNova Dual Source diffractometer with an Atlas detector	4585 independent reflections
Radiation source: SuperNova (Cu) X-ray Source	4078 reflections with *I* > 2σ(*I*)
Mirror monochromator	*R*_int_ = 0.050
Detector resolution: 5.3114 pixels mm^-1^	θ_max_ = 66.9°, θ_min_ = 2.8°
ω scans	*h* = −15→15
Absorption correction: gaussian (*CrysAlis PRO*; Agilent, 2013)	*k* = −7→7
*T*_min_ = 0.899, *T*_max_ = 1.000	*l* = −18→18
19981 measured reflections	

### Refinement

**Table:**

Refinement on *F*^2^	Hydrogen site location: inferred from neighbouring sites
Least-squares matrix: full	H-atom parameters constrained
*R*[*F*^2^ > 2σ(*F*^2^)] = 0.056	*w* = 1/[σ^2^(*F*_o_^2^) + (0.0948*P*)^2^ + 0.4383*P*] where *P* = (*F*_o_^2^ + 2*F*_c_^2^)/3
*wR*(*F*^2^) = 0.150	(Δ/σ)_max_ < 0.001
*S* = 1.06	Δρ_max_ = 0.35 e Å^−^^3^
4585 reflections	Δρ_min_ = −0.23 e Å^−^^3^
341 parameters	Absolute structure: Flack *x* determined using 1671 quotients [(*I*^+^)-(*I*^-^)]/[(*I*^+^)+(*I*^-^)] (Parsons & Flack, 2004)
1 restraint	Absolute structure parameter: 0.09 (14)
Primary atom site location: structure-invariant direct methods	

### Special details

**Table:**

Experimental. Numerical absorption correction based on gaussian integration over a multifaceted crystal model
Geometry. All e.s.d.'s (except the e.s.d. in the dihedral angle between two l.s. planes) are estimated using the full covariance matrix. The cell e.s.d.'s are taken into account individually in the estimation of e.s.d.'s in distances, angles and torsion angles; correlations between e.s.d.'s in cell parameters are only used when they are defined by crystal symmetry. An approximate (isotropic) treatment of cell e.s.d.'s is used for estimating e.s.d.'s involving l.s. planes.

### Fractional atomic coordinates and isotropic or equivalent isotropic displacement parameters (Å^2^)

**Table:**

	*x*	*y*	*z*	*U*_iso_*/*U*_eq_	
O1	0.77480 (17)	0.1640 (3)	1.04104 (13)	0.0330 (5)	
O2	0.64687 (16)	0.3772 (3)	0.97693 (13)	0.0307 (5)	
O3	0.88440 (18)	0.5994 (4)	1.15707 (14)	0.0414 (6)	
O4	0.91050 (17)	0.2056 (3)	0.85758 (13)	0.0316 (5)	
H4	0.9698	0.2203	0.8449	0.047*	
N1	0.61755 (19)	0.3716 (4)	0.58123 (15)	0.0267 (5)	
H1	0.5782	0.2885	0.6050	0.032*	
C1	0.7110 (2)	0.4610 (4)	0.61906 (19)	0.0260 (6)	
C2	0.7906 (2)	0.4099 (4)	0.69719 (18)	0.0252 (6)	
C3	0.7579 (2)	0.4000 (5)	0.78767 (18)	0.0264 (6)	
C4	0.6942 (2)	0.1981 (4)	0.80235 (19)	0.0279 (6)	
H4A	0.6195	0.2288	0.7870	0.033*	
H4B	0.7117	0.0912	0.7637	0.033*	
C5	0.7119 (2)	0.1065 (5)	0.89279 (19)	0.0301 (6)	
H5A	0.6482	0.0338	0.9021	0.036*	
H5B	0.7684	0.0044	0.8968	0.036*	
C6	0.7394 (2)	0.2648 (5)	0.96202 (19)	0.0293 (7)	
C7	0.7658 (3)	0.3273 (5)	1.10039 (19)	0.0327 (7)	
H7	0.7714	0.2729	1.1583	0.039*	
C8	0.8486 (2)	0.4967 (5)	1.0940 (2)	0.0338 (7)	
C9	0.8771 (2)	0.5268 (5)	1.0107 (2)	0.0328 (7)	
H9	0.9297	0.6218	1.0028	0.039*	
C10	0.8270 (2)	0.4165 (5)	0.94420 (19)	0.0287 (6)	
C11	0.8592 (2)	0.4071 (5)	0.85721 (18)	0.0270 (6)	
C12	0.9380 (2)	0.5804 (5)	0.8432 (2)	0.0316 (7)	
H12A	0.9080	0.7144	0.8551	0.038*	
H12B	1.0022	0.5614	0.8834	0.038*	
C13	0.9661 (2)	0.5851 (5)	0.75408 (19)	0.0326 (7)	
H13A	1.0117	0.7032	0.7477	0.039*	
H13B	1.0029	0.4580	0.7430	0.039*	
C14	0.8634 (2)	0.6041 (5)	0.69266 (19)	0.0277 (6)	
H14	0.8256	0.7216	0.7135	0.033*	
C15	0.8636 (2)	0.6488 (5)	0.59791 (19)	0.0313 (7)	
H15A	0.8759	0.7955	0.5876	0.038*	
H15B	0.9157	0.5649	0.5751	0.038*	
C16	0.7522 (2)	0.5841 (4)	0.56234 (19)	0.0276 (6)	
C17	0.6812 (2)	0.5766 (4)	0.48313 (18)	0.0262 (6)	
C18	0.6770 (2)	0.6725 (4)	0.40181 (18)	0.0267 (6)	
C19	0.5951 (2)	0.6126 (5)	0.33970 (19)	0.0310 (7)	
H19	0.5918	0.6687	0.2856	0.037*	
C20	0.5165 (2)	0.4704 (5)	0.35503 (19)	0.0308 (7)	
H20	0.4644	0.4318	0.3105	0.037*	
C21	0.5147 (2)	0.3863 (5)	0.43453 (19)	0.0279 (6)	
H21	0.4605	0.2986	0.4455	0.033*	
C22	0.5987 (2)	0.4402 (4)	0.49762 (18)	0.0262 (6)	
C23	0.8453 (3)	0.2072 (5)	0.6718 (2)	0.0308 (7)	
H23A	0.7937	0.0983	0.6598	0.046*	
H23B	0.8987	0.1648	0.7176	0.046*	
H23C	0.8774	0.2338	0.6223	0.046*	
C24	0.6862 (2)	0.5888 (4)	0.80049 (19)	0.0281 (6)	
H24A	0.7270	0.7150	0.8035	0.042*	
H24B	0.6576	0.5712	0.8523	0.042*	
H24C	0.6294	0.5971	0.7537	0.042*	
C25	0.6530 (2)	0.4108 (6)	1.06813 (19)	0.0336 (7)	
C26	0.6353 (3)	0.6379 (5)	1.0847 (2)	0.0384 (8)	
H26A	0.6817	0.7211	1.0565	0.058*	
H26B	0.6497	0.6639	1.1447	0.058*	
H26C	0.5633	0.6738	1.0637	0.058*	
C27	0.5722 (3)	0.2731 (6)	1.1015 (2)	0.0376 (8)	
H27A	0.5028	0.3075	1.0731	0.056*	
H27B	0.5757	0.2954	1.1614	0.056*	
H27C	0.5872	0.1292	1.0912	0.056*	
C28	0.7593 (2)	0.8372 (4)	0.38782 (18)	0.0295 (7)	
C29	0.7411 (3)	0.9297 (5)	0.2981 (2)	0.0381 (8)	
H29A	0.7483	0.8217	0.2576	0.057*	
H29B	0.7924	1.0372	0.2940	0.057*	
H29C	0.6714	0.9881	0.2866	0.057*	
C30	0.7512 (3)	1.0194 (5)	0.4492 (2)	0.0351 (7)	
H30A	0.6870	1.0954	0.4313	0.053*	
H30B	0.8106	1.1109	0.4494	0.053*	
H30C	0.7508	0.9657	0.5054	0.053*	
C31	0.8695 (3)	0.7438 (5)	0.4004 (2)	0.0340 (7)	
H31	0.9255	0.8328	0.4195	0.041*	
C32	0.8925 (3)	0.5482 (6)	0.3866 (2)	0.0426 (8)	
H32A	0.8388	0.4540	0.3675	0.051*	
H32B	0.9625	0.5039	0.3960	0.051*	

### Atomic displacement parameters (Å^2^)

**Table:**

	*U*^11^	*U*^22^	*U*^33^	*U*^12^	*U*^13^	*U*^23^
O1	0.0397 (12)	0.0260 (10)	0.0297 (11)	0.0012 (9)	−0.0062 (9)	0.0028 (9)
O2	0.0311 (10)	0.0307 (11)	0.0269 (10)	−0.0007 (9)	−0.0065 (8)	−0.0031 (9)
O3	0.0390 (12)	0.0489 (14)	0.0317 (12)	−0.0085 (11)	−0.0090 (10)	−0.0092 (11)
O4	0.0329 (11)	0.0272 (11)	0.0327 (11)	0.0059 (9)	−0.0019 (9)	0.0019 (9)
N1	0.0308 (12)	0.0218 (12)	0.0254 (12)	−0.0015 (10)	−0.0027 (10)	0.0024 (10)
C1	0.0273 (14)	0.0188 (13)	0.0298 (15)	0.0013 (11)	−0.0018 (12)	−0.0022 (11)
C2	0.0296 (14)	0.0163 (12)	0.0270 (14)	0.0012 (12)	−0.0037 (11)	−0.0015 (11)
C3	0.0304 (14)	0.0177 (13)	0.0276 (15)	0.0005 (12)	−0.0062 (12)	−0.0004 (11)
C4	0.0310 (15)	0.0198 (14)	0.0303 (14)	−0.0030 (12)	−0.0031 (12)	−0.0014 (11)
C5	0.0368 (16)	0.0186 (13)	0.0330 (16)	−0.0041 (12)	−0.0006 (13)	−0.0036 (12)
C6	0.0351 (16)	0.0229 (15)	0.0268 (15)	0.0005 (12)	−0.0055 (12)	−0.0014 (11)
C7	0.0348 (16)	0.0349 (16)	0.0250 (15)	−0.0017 (13)	−0.0065 (12)	0.0008 (13)
C8	0.0299 (15)	0.0364 (17)	0.0325 (16)	0.0013 (13)	−0.0034 (13)	−0.0014 (13)
C9	0.0330 (15)	0.0330 (16)	0.0296 (15)	−0.0062 (13)	−0.0044 (12)	−0.0008 (13)
C10	0.0307 (14)	0.0212 (14)	0.0314 (15)	0.0026 (12)	−0.0038 (12)	0.0015 (12)
C11	0.0281 (14)	0.0216 (13)	0.0286 (15)	0.0006 (12)	−0.0044 (12)	−0.0014 (12)
C12	0.0316 (15)	0.0278 (15)	0.0325 (16)	−0.0030 (13)	−0.0047 (12)	−0.0014 (13)
C13	0.0306 (15)	0.0311 (15)	0.0330 (16)	−0.0027 (13)	−0.0055 (13)	−0.0035 (13)
C14	0.0285 (14)	0.0231 (14)	0.0290 (15)	−0.0015 (12)	−0.0037 (12)	0.0008 (12)
C15	0.0386 (17)	0.0234 (14)	0.0283 (15)	−0.0022 (12)	−0.0056 (13)	0.0026 (12)
C16	0.0322 (14)	0.0193 (13)	0.0292 (15)	0.0002 (12)	−0.0021 (12)	−0.0022 (12)
C17	0.0307 (14)	0.0206 (13)	0.0260 (14)	0.0018 (12)	−0.0001 (12)	−0.0008 (11)
C18	0.0329 (15)	0.0207 (13)	0.0247 (14)	−0.0001 (12)	−0.0007 (11)	0.0007 (11)
C19	0.0353 (15)	0.0265 (15)	0.0287 (15)	0.0036 (13)	−0.0034 (13)	0.0026 (12)
C20	0.0329 (15)	0.0247 (14)	0.0300 (15)	0.0019 (12)	−0.0099 (13)	−0.0009 (12)
C21	0.0293 (14)	0.0200 (13)	0.0318 (15)	−0.0002 (12)	−0.0032 (12)	0.0019 (12)
C22	0.0327 (15)	0.0190 (14)	0.0254 (14)	0.0028 (11)	−0.0006 (12)	−0.0004 (11)
C23	0.0365 (16)	0.0212 (15)	0.0326 (15)	0.0024 (12)	−0.0016 (13)	−0.0028 (12)
C24	0.0362 (16)	0.0183 (13)	0.0280 (14)	0.0027 (12)	−0.0013 (12)	−0.0020 (11)
C25	0.0339 (16)	0.0382 (17)	0.0255 (15)	−0.0009 (14)	−0.0059 (12)	−0.0034 (13)
C26	0.0408 (17)	0.0387 (18)	0.0317 (16)	0.0011 (14)	−0.0074 (13)	−0.0077 (14)
C27	0.0336 (17)	0.0419 (19)	0.0354 (17)	−0.0041 (14)	−0.0010 (14)	−0.0025 (14)
C28	0.0384 (16)	0.0222 (15)	0.0262 (15)	−0.0027 (12)	−0.0007 (12)	0.0031 (12)
C29	0.0478 (19)	0.0319 (17)	0.0310 (16)	−0.0070 (14)	−0.0054 (14)	0.0056 (13)
C30	0.0453 (18)	0.0211 (15)	0.0359 (17)	−0.0015 (13)	−0.0034 (14)	0.0009 (13)
C31	0.0332 (16)	0.0319 (16)	0.0349 (16)	−0.0061 (13)	−0.0013 (13)	0.0006 (13)
C32	0.0397 (18)	0.0356 (18)	0.052 (2)	0.0042 (15)	0.0068 (16)	0.0025 (15)

### Geometric parameters (Å, º)

**Table:**

O1—C7	1.427 (4)	C15—C16	1.509 (4)
O1—C6	1.427 (4)	C15—H15A	0.9700
O2—C6	1.437 (4)	C15—H15B	0.9700
O2—C25	1.461 (4)	C16—C17	1.440 (4)
O3—C8	1.231 (4)	C17—C22	1.417 (4)
O4—C11	1.447 (4)	C17—C18	1.429 (4)
O4—H4	0.8200	C18—C19	1.382 (4)
N1—C1	1.379 (4)	C18—C28	1.532 (4)
N1—C22	1.390 (4)	C19—C20	1.406 (5)
N1—H1	0.8600	C19—H19	0.9300
C1—C16	1.366 (4)	C20—C21	1.381 (4)
C1—C2	1.520 (4)	C20—H20	0.9300
C2—C23	1.557 (4)	C21—C22	1.399 (4)
C2—C14	1.562 (4)	C21—H21	0.9300
C2—C3	1.565 (4)	C23—H23A	0.9600
C3—C24	1.550 (4)	C23—H23B	0.9600
C3—C4	1.565 (4)	C23—H23C	0.9600
C3—C11	1.573 (4)	C24—H24A	0.9600
C4—C5	1.542 (4)	C24—H24B	0.9600
C4—H4A	0.9700	C24—H24C	0.9600
C4—H4B	0.9700	C25—C26	1.501 (5)
C5—C6	1.500 (4)	C25—C27	1.517 (5)
C5—H5A	0.9700	C26—H26A	0.9600
C5—H5B	0.9700	C26—H26B	0.9600
C6—C10	1.543 (4)	C26—H26C	0.9600
C7—C8	1.531 (5)	C27—H27A	0.9600
C7—C25	1.552 (4)	C27—H27B	0.9600
C7—H7	0.9800	C27—H27C	0.9600
C8—C9	1.443 (5)	C28—C31	1.516 (4)
C9—C10	1.351 (4)	C28—C29	1.533 (4)
C9—H9	0.9300	C28—C30	1.537 (4)
C10—C11	1.508 (4)	C29—H29A	0.9600
C11—C12	1.538 (4)	C29—H29B	0.9600
C12—C13	1.521 (4)	C29—H29C	0.9600
C12—H12A	0.9700	C30—H30A	0.9600
C12—H12B	0.9700	C30—H30B	0.9600
C13—C14	1.520 (4)	C30—H30C	0.9600
C13—H13A	0.9700	C31—C32	1.313 (5)
C13—H13B	0.9700	C31—H31	0.9300
C14—C15	1.539 (4)	C32—H32A	0.9300
C14—H14	0.9800	C32—H32B	0.9300
			
C7—O1—C6	102.0 (2)	C14—C15—H15A	111.8
C6—O2—C25	108.6 (2)	C16—C15—H15B	111.8
C11—O4—H4	109.5	C14—C15—H15B	111.8
C1—N1—C22	107.5 (2)	H15A—C15—H15B	109.5
C1—N1—H1	126.3	C1—C16—C17	107.9 (3)
C22—N1—H1	126.3	C1—C16—C15	110.4 (3)
C16—C1—N1	110.3 (2)	C17—C16—C15	140.5 (3)
C16—C1—C2	112.9 (2)	C22—C17—C18	119.1 (3)
N1—C1—C2	134.2 (3)	C22—C17—C16	105.3 (2)
C1—C2—C23	103.7 (2)	C18—C17—C16	135.6 (3)
C1—C2—C14	96.9 (2)	C19—C18—C17	116.8 (3)
C23—C2—C14	110.8 (2)	C19—C18—C28	123.4 (3)
C1—C2—C3	121.6 (2)	C17—C18—C28	119.8 (2)
C23—C2—C3	113.7 (2)	C18—C19—C20	122.6 (3)
C14—C2—C3	108.7 (2)	C18—C19—H19	118.7
C24—C3—C2	109.7 (2)	C20—C19—H19	118.7
C24—C3—C4	106.9 (2)	C21—C20—C19	121.7 (3)
C2—C3—C4	113.0 (2)	C21—C20—H20	119.2
C24—C3—C11	109.0 (2)	C19—C20—H20	119.2
C2—C3—C11	109.9 (2)	C20—C21—C22	116.5 (3)
C4—C3—C11	108.1 (2)	C20—C21—H21	121.8
C5—C4—C3	116.7 (2)	C22—C21—H21	121.8
C5—C4—H4A	108.1	N1—C22—C21	127.9 (3)
C3—C4—H4A	108.1	N1—C22—C17	109.1 (2)
C5—C4—H4B	108.1	C21—C22—C17	123.0 (3)
C3—C4—H4B	108.1	C2—C23—H23A	109.5
H4A—C4—H4B	107.3	C2—C23—H23B	109.5
C6—C5—C4	114.6 (2)	H23A—C23—H23B	109.5
C6—C5—H5A	108.6	C2—C23—H23C	109.5
C4—C5—H5A	108.6	H23A—C23—H23C	109.5
C6—C5—H5B	108.6	H23B—C23—H23C	109.5
C4—C5—H5B	108.6	C3—C24—H24A	109.5
H5A—C5—H5B	107.6	C3—C24—H24B	109.5
O1—C6—O2	103.8 (2)	H24A—C24—H24B	109.5
O1—C6—C5	110.6 (2)	C3—C24—H24C	109.5
O2—C6—C5	110.7 (2)	H24A—C24—H24C	109.5
O1—C6—C10	107.8 (2)	H24B—C24—H24C	109.5
O2—C6—C10	110.9 (2)	O2—C25—C26	109.4 (3)
C5—C6—C10	112.6 (3)	O2—C25—C27	109.2 (3)
O1—C7—C8	110.3 (3)	C26—C25—C27	111.8 (3)
O1—C7—C25	101.4 (2)	O2—C25—C7	100.7 (2)
C8—C7—C25	110.6 (3)	C26—C25—C7	115.7 (3)
O1—C7—H7	111.4	C27—C25—C7	109.5 (3)
C8—C7—H7	111.4	C25—C26—H26A	109.5
C25—C7—H7	111.4	C25—C26—H26B	109.5
O3—C8—C9	124.6 (3)	H26A—C26—H26B	109.5
O3—C8—C7	119.9 (3)	C25—C26—H26C	109.5
C9—C8—C7	115.5 (3)	H26A—C26—H26C	109.5
C10—C9—C8	119.8 (3)	H26B—C26—H26C	109.5
C10—C9—H9	120.1	C25—C27—H27A	109.5
C8—C9—H9	120.1	C25—C27—H27B	109.5
C9—C10—C11	125.4 (3)	H27A—C27—H27B	109.5
C9—C10—C6	117.1 (3)	C25—C27—H27C	109.5
C11—C10—C6	116.9 (3)	H27A—C27—H27C	109.5
O4—C11—C10	102.7 (2)	H27B—C27—H27C	109.5
O4—C11—C12	109.6 (2)	C31—C28—C18	110.9 (2)
C10—C11—C12	112.5 (2)	C31—C28—C29	106.3 (3)
O4—C11—C3	107.6 (2)	C18—C28—C29	113.1 (3)
C10—C11—C3	109.8 (2)	C31—C28—C30	111.6 (3)
C12—C11—C3	114.0 (2)	C18—C28—C30	108.3 (2)
C13—C12—C11	113.9 (2)	C29—C28—C30	106.7 (3)
C13—C12—H12A	108.8	C28—C29—H29A	109.5
C11—C12—H12A	108.8	C28—C29—H29B	109.5
C13—C12—H12B	108.8	H29A—C29—H29B	109.5
C11—C12—H12B	108.8	C28—C29—H29C	109.5
H12A—C12—H12B	107.7	H29A—C29—H29C	109.5
C14—C13—C12	107.4 (2)	H29B—C29—H29C	109.5
C14—C13—H13A	110.2	C28—C30—H30A	109.5
C12—C13—H13A	110.2	C28—C30—H30B	109.5
C14—C13—H13B	110.2	H30A—C30—H30B	109.5
C12—C13—H13B	110.2	C28—C30—H30C	109.5
H13A—C13—H13B	108.5	H30A—C30—H30C	109.5
C13—C14—C15	121.1 (3)	H30B—C30—H30C	109.5
C13—C14—C2	111.7 (2)	C32—C31—C28	125.6 (3)
C15—C14—C2	106.5 (2)	C32—C31—H31	117.2
C13—C14—H14	105.4	C28—C31—H31	117.2
C15—C14—H14	105.4	C31—C32—H32A	120.0
C2—C14—H14	105.4	C31—C32—H32B	120.0
C16—C15—C14	100.1 (2)	H32A—C32—H32B	120.0
C16—C15—H15A	111.8		
			
C22—N1—C1—C16	0.0 (3)	C4—C3—C11—C12	171.7 (2)
C22—N1—C1—C2	−159.8 (3)	O4—C11—C12—C13	70.5 (3)
C16—C1—C2—C23	−88.8 (3)	C10—C11—C12—C13	−175.9 (2)
N1—C1—C2—C23	70.7 (4)	C3—C11—C12—C13	−50.1 (3)
C16—C1—C2—C14	24.7 (3)	C11—C12—C13—C14	55.7 (3)
N1—C1—C2—C14	−175.9 (3)	C12—C13—C14—C15	170.0 (3)
C16—C1—C2—C3	141.7 (3)	C12—C13—C14—C2	−63.3 (3)
N1—C1—C2—C3	−58.8 (4)	C1—C2—C14—C13	−169.4 (2)
C1—C2—C3—C24	−44.3 (3)	C23—C2—C14—C13	−61.8 (3)
C23—C2—C3—C24	−169.4 (2)	C3—C2—C14—C13	63.8 (3)
C14—C2—C3—C24	66.6 (3)	C1—C2—C14—C15	−35.2 (3)
C1—C2—C3—C4	74.9 (3)	C23—C2—C14—C15	72.4 (3)
C23—C2—C3—C4	−50.1 (3)	C3—C2—C14—C15	−162.0 (2)
C14—C2—C3—C4	−174.1 (2)	C13—C14—C15—C16	162.6 (3)
C1—C2—C3—C11	−164.2 (2)	C2—C14—C15—C16	33.6 (3)
C23—C2—C3—C11	70.7 (3)	N1—C1—C16—C17	0.6 (3)
C14—C2—C3—C11	−53.2 (3)	C2—C1—C16—C17	165.0 (2)
C24—C3—C4—C5	−93.2 (3)	N1—C1—C16—C15	−169.2 (2)
C2—C3—C4—C5	145.9 (3)	C2—C1—C16—C15	−4.8 (3)
C11—C3—C4—C5	24.0 (3)	C14—C15—C16—C1	−18.0 (3)
C3—C4—C5—C6	30.1 (4)	C14—C15—C16—C17	177.3 (4)
C7—O1—C6—O2	−44.4 (3)	C1—C16—C17—C22	−0.9 (3)
C7—O1—C6—C5	−163.2 (3)	C15—C16—C17—C22	164.0 (4)
C7—O1—C6—C10	73.3 (3)	C1—C16—C17—C18	177.8 (3)
C25—O2—C6—O1	22.5 (3)	C15—C16—C17—C18	−17.3 (6)
C25—O2—C6—C5	141.2 (2)	C22—C17—C18—C19	−4.7 (4)
C25—O2—C6—C10	−93.0 (3)	C16—C17—C18—C19	176.7 (3)
C4—C5—C6—O1	−168.4 (2)	C22—C17—C18—C28	173.7 (3)
C4—C5—C6—O2	77.1 (3)	C16—C17—C18—C28	−4.8 (5)
C4—C5—C6—C10	−47.7 (3)	C17—C18—C19—C20	2.3 (4)
C6—O1—C7—C8	−69.4 (3)	C28—C18—C19—C20	−176.1 (3)
C6—O1—C7—C25	47.9 (3)	C18—C19—C20—C21	2.1 (5)
O1—C7—C8—O3	−149.9 (3)	C19—C20—C21—C22	−3.8 (4)
C25—C7—C8—O3	98.7 (3)	C1—N1—C22—C21	178.2 (3)
O1—C7—C8—C9	31.6 (4)	C1—N1—C22—C17	−0.6 (3)
C25—C7—C8—C9	−79.8 (3)	C20—C21—C22—N1	−177.4 (3)
O3—C8—C9—C10	−174.8 (3)	C20—C21—C22—C17	1.2 (4)
C7—C8—C9—C10	3.5 (4)	C18—C17—C22—N1	−178.0 (3)
C8—C9—C10—C11	−170.2 (3)	C16—C17—C22—N1	0.9 (3)
C8—C9—C10—C6	1.0 (4)	C18—C17—C22—C21	3.1 (4)
O1—C6—C10—C9	−40.5 (4)	C16—C17—C22—C21	−178.0 (3)
O2—C6—C10—C9	72.5 (3)	C6—O2—C25—C26	128.7 (3)
C5—C6—C10—C9	−162.8 (3)	C6—O2—C25—C27	−108.7 (3)
O1—C6—C10—C11	131.5 (3)	C6—O2—C25—C7	6.5 (3)
O2—C6—C10—C11	−115.5 (3)	O1—C7—C25—O2	−33.0 (3)
C5—C6—C10—C11	9.2 (4)	C8—C7—C25—O2	84.1 (3)
C9—C10—C11—O4	102.7 (3)	O1—C7—C25—C26	−150.7 (3)
C6—C10—C11—O4	−68.5 (3)	C8—C7—C25—C26	−33.7 (4)
C9—C10—C11—C12	−15.1 (4)	O1—C7—C25—C27	81.9 (3)
C6—C10—C11—C12	173.7 (2)	C8—C7—C25—C27	−161.0 (3)
C9—C10—C11—C3	−143.1 (3)	C19—C18—C28—C31	−118.9 (3)
C6—C10—C11—C3	45.7 (3)	C17—C18—C28—C31	62.7 (3)
C24—C3—C11—O4	165.8 (2)	C19—C18—C28—C29	0.4 (4)
C2—C3—C11—O4	−73.9 (3)	C17—C18—C28—C29	−178.0 (3)
C4—C3—C11—O4	49.9 (3)	C19—C18—C28—C30	118.4 (3)
C24—C3—C11—C10	54.8 (3)	C17—C18—C28—C30	−60.0 (3)
C2—C3—C11—C10	175.1 (2)	C18—C28—C31—C32	29.3 (4)
C4—C3—C11—C10	−61.1 (3)	C29—C28—C31—C32	−94.0 (4)
C24—C3—C11—C12	−72.4 (3)	C30—C28—C31—C32	150.1 (3)
C2—C3—C11—C12	47.9 (3)		

### Hydrogen-bond geometry (Å, º)

Cg is the centroid of the C17–C22 ring.

**Table:**

*D*—H···*A*	*D*—H	H···*A*	*D*···*A*	*D*—H···*A*
O4—H4···O3^i^	0.82	2.03	2.757 (3)	148
N1—H1···*Cg*^ii^	0.86	2.78	3.527 (1)	146

Symmetry codes: (i) −*x*+2, *y*−1/2, −*z*+2; (ii) −*x*+1, *y*−1/2, −*z*+1.
