# Supplementary figures and images for: Crystal and mol­ecular structure of aflatrem
Source: Acta Crystallogr E Crystallogr Commun. 2015 Oct 17;71(Pt 11):o867–8. doi: 10.1107/S2056989015019040 (PMC4645010; doi:10.1107/S2056989015019040)

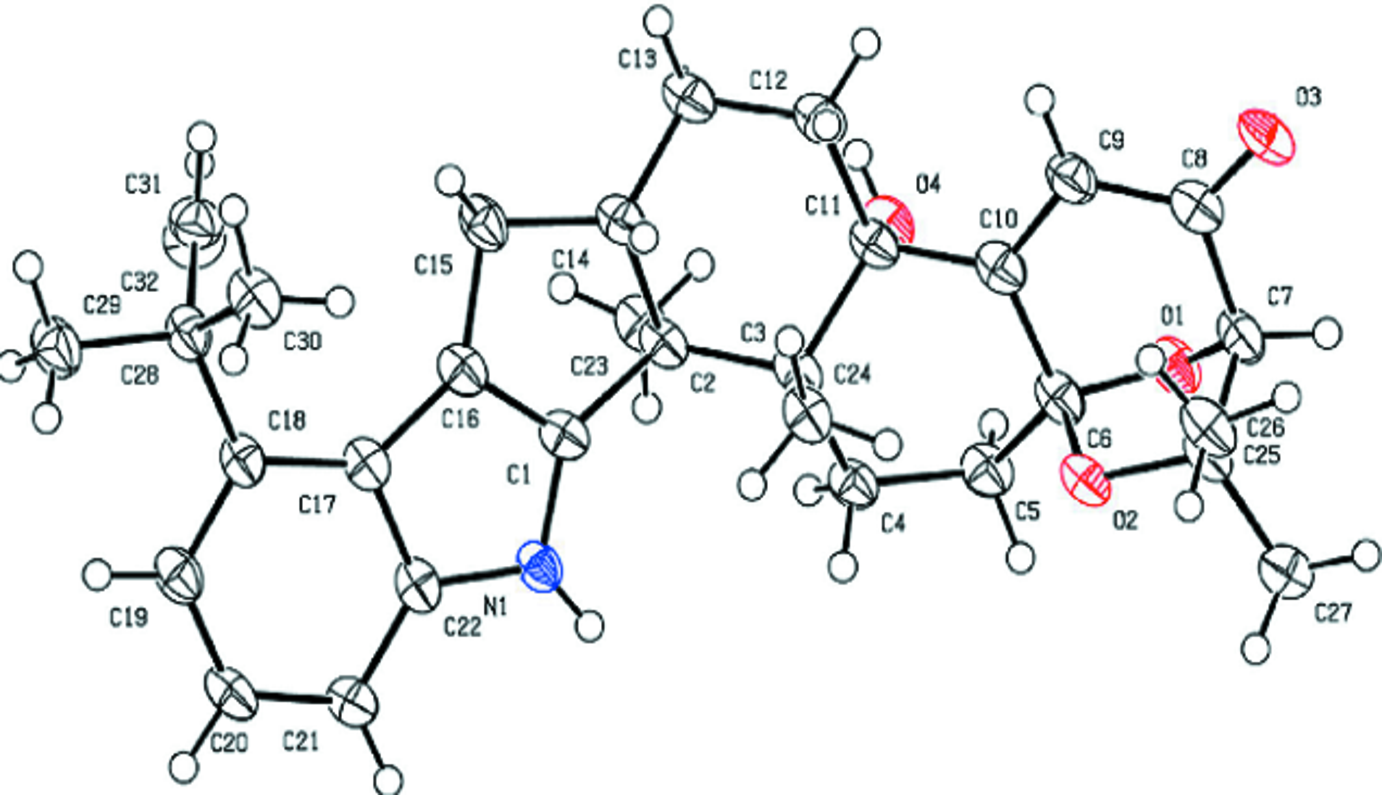

Supplement: Supplementary file 3 [file e-71-0o867-fig1.tif]

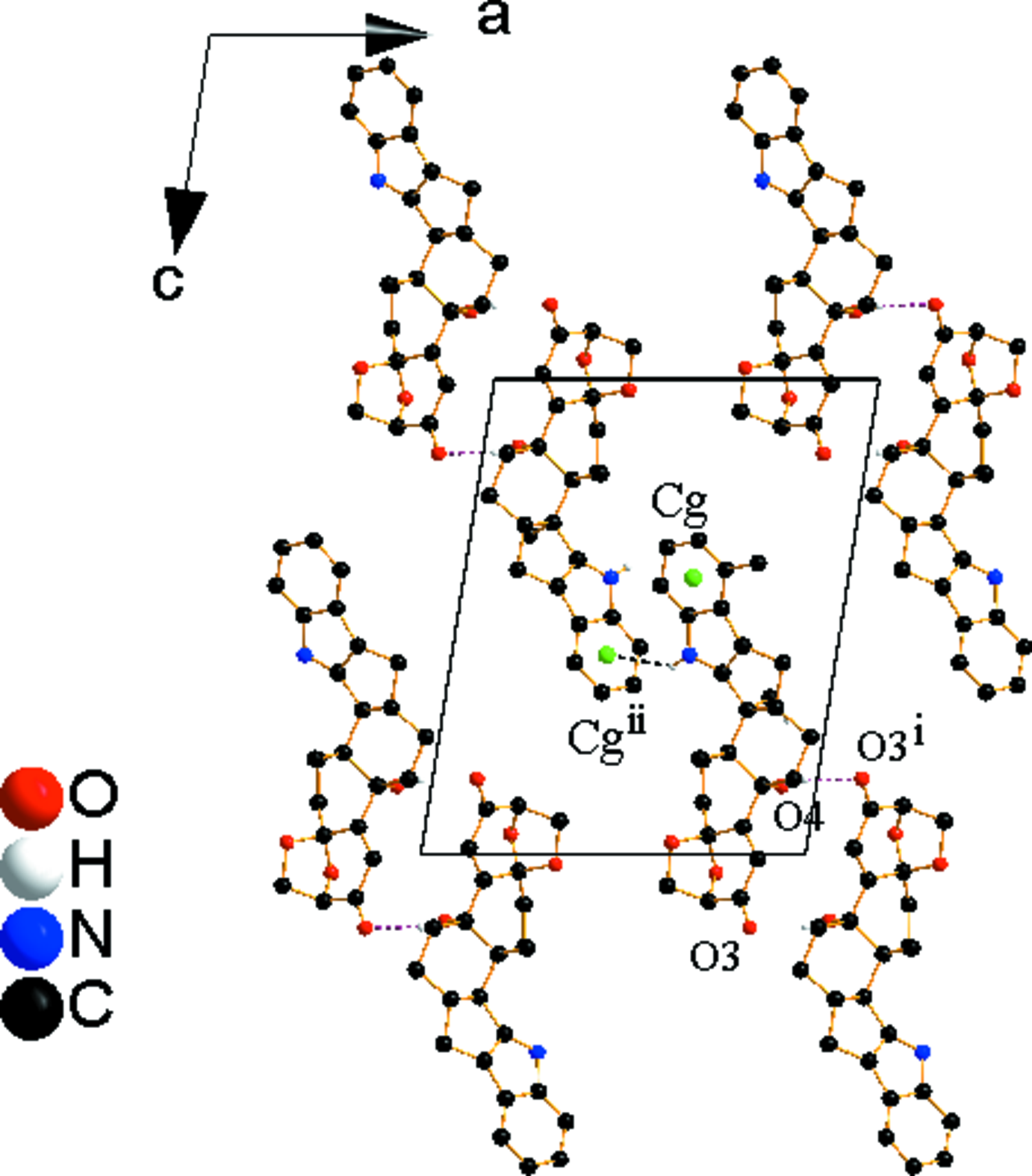

Supplement: Supplementary file 4 [file e-71-0o867-fig2.tif]

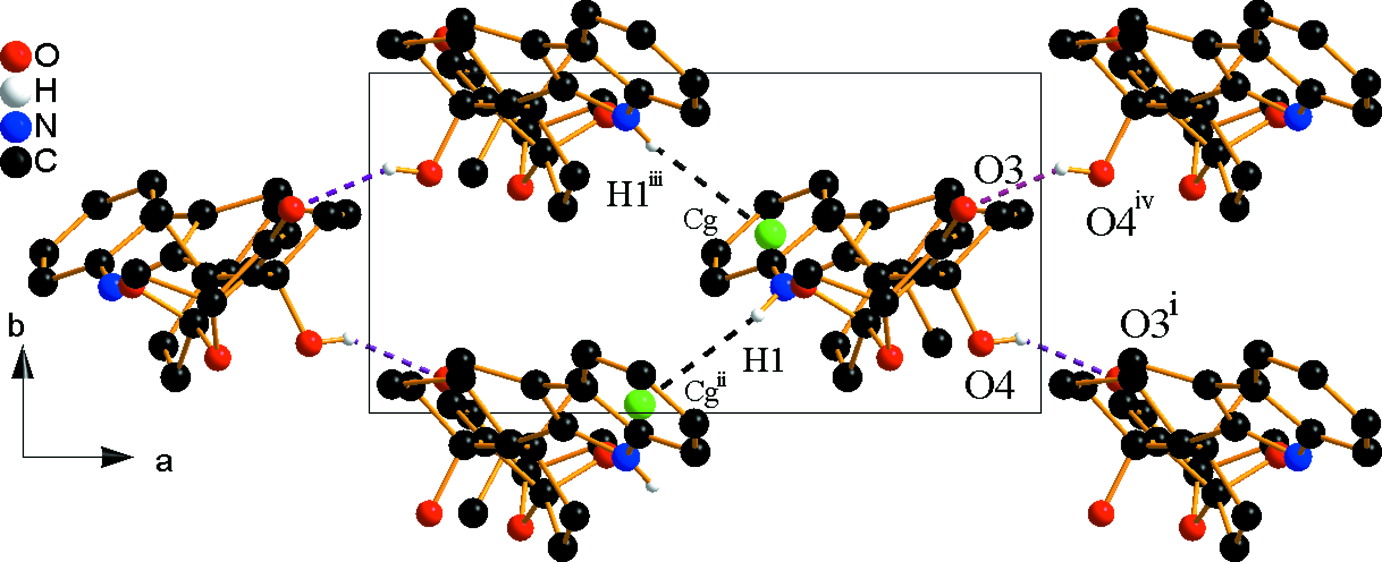

Supplement: Supplementary file 5 [file e-71-0o867-fig3.tif]
